# Quantitative methylation analysis reveals distinct association between *PAX6* methylation and clinical characteristics with different viral infections in hepatocellular carcinoma

**DOI:** 10.1186/s13148-016-0208-3

**Published:** 2016-04-22

**Authors:** Yu-Lueng Shih, Chih-Chi Kuo, Ming-De Yan, Ya-Wen Lin, Chung-Bao Hsieh, Tsai-Yuan Hsieh

**Affiliations:** Division of Gastroenterology, Department of Internal Medicine, Tri-Service General Hospital, National Defense Medical Center, Taipei, Taiwan, Republic of China; Department and Graduate Institute of Microbiology and Immunology, National Defense Medical Center, Taipei, Taiwan, Republic of China; Cancer Center, Wan Fang Hospital, Taipei Medical University, Taipei, Republic of China; Graduate Institute of Life Sciences, National Defense Medical Center, Taipei, Taiwan, Republic of China; Division of General Surgery, Department of Surgery, Tri-Service General Hospital, National Defense Medical Center, Taipei, Taiwan, Republic of China

**Keywords:** Hepatocellular carcinoma, DNA methylation, Q-MSP, *PAX6*, HCV

## Abstract

**Background:**

Related to genetic alteration, frequent promoter hypermethylation is also a contributing factor in the development of human cancers. Recently, we discovered numerous novel genes that were aberrantly methylated in hepatocellular carcinoma (HCC) by using Infinium HumanMethylation27 BeadChip array. We utilized a quantitative methylation-specific PCR (Q-MSP) system for the evaluation of *PAX6* methylation in 29 normal controls and 160 paired HCC tissues and their adjacent non-tumor tissues. We verified the correlation between the methylation status of *PAX6* and clinical characteristics with different viral status.

**Results:**

*Paired-box 6* promoter methylation was observed in 39.4 %, 15.6 %, and 3.4 % in primary HCCs, adjacent non-tumors, and normal control tissues, respectively. Methylation of the *PAX6* promoter region in HCCs significantly increased compared with control tissues. *PAX6* was frequently methylated in HCV-positive HCC tissues (61.3 %) and rarely methylated in HBV-positive (22.1 %) and double-negative HCC tissues (33.3 %).

**Conclusions:**

Our data suggests that promoter hypermethylation of *PAX6* is a common event in HCCs and the association of *PAX6* methylation in clinicopathological features is divergent with different viral status.

**Electronic supplementary material:**

The online version of this article (doi:10.1186/s13148-016-0208-3) contains supplementary material, which is available to authorized users.

## Background

Hepatocellular carcinoma (HCC) is one of the most malignant tumors worldwide [1, 2]. It is a fatal disease due to difficulties in early detection which leads to poor prognosis and high mortality rates. Currently, therapeutic options are not effective, as indicated by data results for reaching a global survival rate of 20–65 % for 1 year, 10–30 % for 3 years, and 10–20 % for 5 years after diagnosis [3, 4]. The most significant risk factors associated with HCC include chronic hepatitis B and C virus (HBV and HCV) infections, cryptogenic liver cirrhosis, aflatoxin-B1-contaminated food, excessive alcohol consumption, and obesity [1]. The recent increase in the incidence of HCC in Western countries is predominantly due to the HCV endemic and the lack of effective vaccination. Despite significant effort made over the past decade to understand the development of HCC, the genetic alterations that lead to the initiation and progression of HCC still remain largely unknown. In addition to genetic alterations, epigenetic alterations have been progressively recognized as a key impetus for carcinogenesis. Methylation of CpG islands in promoter regions is frequently associated with transcriptional silencing and is implicated in tumor suppressor gene (TSG) inactivation in cancer cells [5]. Some studies have investigated the methylation profiles associated with different viral infections but without the yield of any definitive conclusions.

Recently, technical advances in array systems, such as the Infinium assay [6] can detect altered methylation patterns and other epigenetic changes in cancer. It has also been successfully applied in the study of HCC [7–10]. An advantage of adapting array-based platforms is that researchers can analyze DNA methylation of regions with no prior knowledge of the sequence. Furthermore, by validating the results from the high-throughput screening approach, researchers can effectively discover more novel genes that may have potential applications in clinical practice.

In our recent analysis [11], we recognized several aberrantly methylated genes in HCC by using the Infinium HumanMethylation27 BeadChip and then verified 34 genes by methylation-specific PCR (MSP). Of these genes, we further proved that frequent methylation of homeobox A9 (*HOXA9*) in HCC tissues and plasma samples from patients could be a useful biomarker to assist in HCC detection. *IRAK3* methylation was associated with tumor stage and poor prognosis of patients [12]. However, several novel genes in our array data were not further validated by quantitative MSP (Q-MSP), such as *PAX6*. Paired-box 6 (PAX6), located on chromosome 11p13, a gene coding for a DNA binding/transcription control protein, participates in normal eye, nose, pancreas, and brain development [13]. *PAX6* exerts a tumor suppressor effect and is frequently silenced by promoter methylation in human cancers, including bladder, breast, gastric, and non-small cell lung cancer [14–17], suggesting that it may play a role in carcinogenesis. Until now, there are no data regarding the *PAX6* methylation in HCC. Moreover, there are no quantitative data about the methylation levels of *PAX6* in HCC.

In the present study, the hypermethylation of CpG islands (CGI) of *PAX6* was quantitatively investigated in HCC by Q-MSP, with particular attention made to the changes in methylation intensities in primary HCC tissues and their corresponding non-tumor liver tissues. The correlation between the methylation status of *PAX6* and clinical disease was also addressed. Finally, we analyzed the promoter methylation level of *PAX6* to be significantly higher in HCV-positive HCC.

## Results

### Distinct DNA methylation patterns and the association with diseases in HCC with different viral infections

**Fig. 1:**
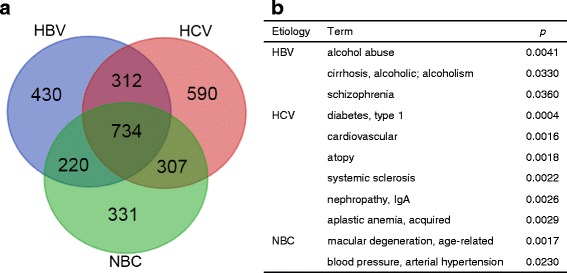
Genes and the association of genes with diseases in HCC with different etiologic factors. The Venn diagram drawing the unique and overlapping genes in HBV-positive (*HBV*), HCV-positive (*HCV*), and double-negative (*NBC*) HCC (**a**). DAVID ontology (disease) for genes abnormally methylated in HBV, HCV, and NBC-HCC (**b**)

In our recent study, we analyzed the methylation profiles of HCC with three different viral etiologies (HBV-positive, HCV-positive, and double-negative) by using the Infinium HumanMethylation27 BeadChip and reported 2924 genes which were aberrantly methylated in non-tumor or tumor tissues of HCC as compared with the normal controls [11]. To further investigate the DNA methylation profiles of unique etiologic-driven HCC, a Venn diagram was utilized to compare the genes identified in each of the etiological groups. Among the 2924 genes, 430, 590, and 331 genes are shown to be aberrantly methylated in HBV-positive, HCV-positive, and double-negative non-tumor or tumor tissues of HCC, respectively (Fig. 1a; Additional file 1: Table S1). To better understand the association between the etiologic-driven changes in DNA methylation patterns and diseases, DAVID disease analysis was used for genes in each etiology. Genes changing in HBV-positive HCC were primarily found to be involved in alcoholic diseases and cirrhosis. Genes identified in HCV-positive HCC were relative to immune deficiency diseases, including type 1 diabetes, atopy, systemic sclerosis, and cardiovascular diseases. Genes in the HCC without hepatitis B and hepatitis C showed the association with age-associated diseases and metabolic diseases (Fig. 1b; Additional file 2: Table S2). Then, to investigate the interest in genes that the changes in DNA methylation status are driven by specific risk factors, the *PAX6* gene was selected as our hypermethylated candidate gene for validation based on the results of the methylation array (data not shown).

### *PAX6* methylation is associated with altered *mRNA* expression in HCC cell lines

**Fig. 2:**
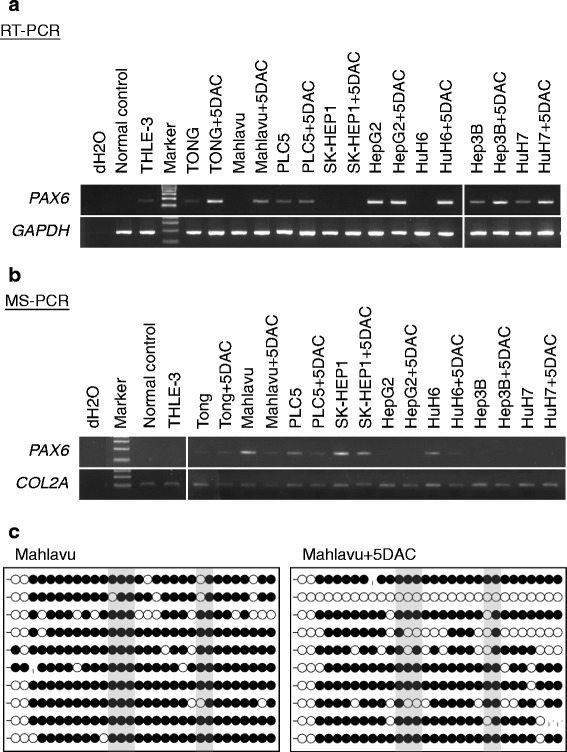
Expression analysis and methylation analysis of *PAX6* in cell lines. *PAX6* and *GAPDH* expression were analyzed by the RT-PCR in the normal control, the normal liver cell line THLE-3, and the HCC cell lines treated with or without DNMT inhibitors (5-aza-2′-deoxycytidine; *5DAC*) (**a**). Promoter methylation of *PAX6* and *COL2A* was analyzed by MS-PCR with methylation-specific primers in the normal control, THLE-3, and HCC cell lines treated with or without 5DAC. CpG methylated human genomic DNA and DNA extracted from normal peripheral blood lymphocytes (PBL) which were modified by sodium bisulfite to generate a positive control, 1/5 diluted positive control, 1/20 diluted positive control, and a negative control, respectively (**b**). *PAX6* methylation was also analyzed by bisulfite sequencing in Mahlavu cells treated with or without 5DAC. Each clone is represented by a *row*, and 30 CpG sites are represented as *circles. Black and white circles* represent methylated cytosine and unmethylated cytosine, respectively. *Gray regions* indicate the CpG sites that the MS-PCR/Q-MSP primer set covered (**c**)

We first investigated the *PAX6* expression in nine HCC cell lines. The reverse transcription polymerase chain reaction (RT-PCR) data showed that there was down-regulation of *PAX6* messenger RNA (mRNA) in HCC cell lines with *PAX6* hypermethylation (Fig. 2a). To confirm that the lack of expression of *PAX6* mRNA in the HCC lines was due to promoter hypermethylation, we treated cells with 5-aza-2′-deoxycytidine (5DAC), an inhibitor of DNA methylation. After treatment with 5 μM of 5DAC, the unmethylated promoter DNA was detected by MSP and bisulfite sequencing (Fig. 2b, c); *PAX6* mRNA was restored or increased in HCC cell lines (Fig. 2a). These data indicate that hypermethylation of *PAX6* may be responsible for the absence or down-regulation of mRNA transcription.

### Frequent methylation of the *PAX6* gene in HCC tissues

**Fig. 3:**
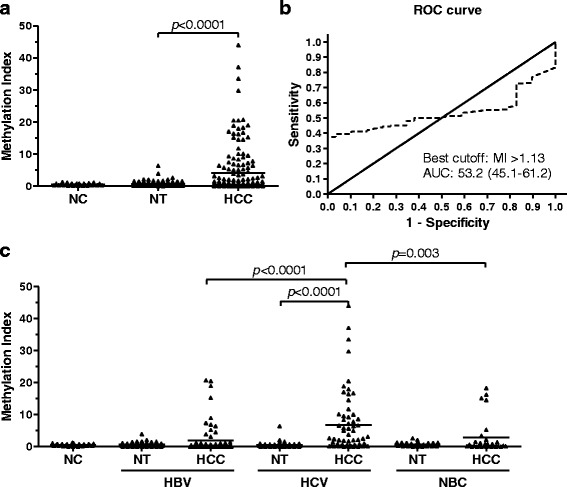
Methylation levels and ROC curve analysis of PAX6 in liver tissues. Gene methylation was determined in 29 normal controls (*NC*) and 160 paired hepatocellular carcinoma (*HCC*) tissues and their adjacent non-tumor tissues (*NT*) by Q-MSP. The results are represented as the difference in the methylation index. The *black lines* indicate the mean of the methylation index. The *p* value for the methylation levels among the groups was computed from Wilcoxon rank-sum test (NC vs HCC and NC vs NT) and Wilcoxon signed-rank test (NT vs HCC) (**a**). The best cutoff value for the methylation index (*MI*) and the area under the receiver operating characteristic curve (*AUC*) were calculated to discriminate 29 normal controls and 160 HCC (**b**). Gene methylation was respectively determined in normal controls (*NC*) and paired hepatocellular carcinoma (*HCC*) tissues and their adjacent non-tumor tissues (*NT*) by etiologic factors. The *p* value for methylation levels among the groups was computed from Wilcoxon rank-sum test (NC vs HCC and NC vs NT) and Wilcoxon signed-rank test (NT vs HCC) (**c**)

The methylation status of the *PAX6* gene was analyzed by Q-MSP in 349 liver tissues, including 29 normal controls and 160 pairs of HCC tissues and their adjacent non-tumor tissues. The results showed a significantly increased methylation level in HCC tissues as compared with non-tumor tissues (*p* < 0.0001) (Fig. 3a). By using receiver operating characteristic (ROC) curve analysis to discriminate 160 HCC tissues and 29 normal controls, the best cutoff value of *PAX6* methylation was MI > 1.13. (Fig. 3b). Under the best cutoff value, 3.4 % of 29 normal controls, 15.6 % of 160 non-tumor tissues, and 39.4 % of 160 HCC tissues were reported as positive for *PAX6* methylation (*p* < 0.0001) (Table 2). An association between gene methylation and clinicopathological features was also analyzed among the 160 HCC patients. Interestingly, there was a statistically positive association between *PAX6* methylation and HCV infection (OR = 4.46; 95 % CI = 2.26–8.80). There was a statistically negative association between *PAX6* methylation and HBV infection (OR = 0.29; 95 % CI = 0.15–0.58), as well as no significance in the association with other clinical parameters (Table 3).

### The association of *PAX6* methylation with clinicopathological features in HCC with different viral status

The methylation status and methylation frequency of the *PAX6* gene was further examined in HCC with different viral etiologies. To test whether the *PAX6* methylation in HCC tissues was associated with different virus infections, we analyzed the methylation level in 29 normal controls (NC) and 160 paired HCC tissues and their adjacent non-tumor tissues. Among the 68 pairs of HCC tissues with HBV infection, 62 pairs exhibited HCV infection and 30 pairs showed neither HBV nor HCV infections. The methylation levels of the *PAX6* gene were significantly increased in HCV-positive HCC tissues (*p* < 0.0001) as compared with their adjacent non-tumor tissues (Fig. 3c). Moreover, the *PAX6* methylation level was significantly higher in HCV-positive HCC as compared with HBV-positive (*p* < 0.0001) and double-negative HCC tissues (*p* = 0.003). Under the best cutoff value (MI > 1.13), *PAX6* was frequently methylated in HCV-positive HCC tissues (61.3 %) and rarely methylated in HBV-positive (22.1 %) and double-negative HCC tissues (33.3 %) (Table 2). In addition, logistic regression analysis showed *PAX6* methylation was not associated with any clinical characteristics in HCV-positive HCC, but it was positively associated with cirrhosis in double-negative HCC tissues (OR = 12.00; 95 % CI = 1.11–129.42) (Table 3).

## Discussion

Epigenetic modifications include DNA methylation, and covalent modification of histones, including methylation, acetylation, and phosphorylation. Alterations in DNA methylation affect the structure of DNA and do not materially affect the genetic code. Methylation of CpG islands in gene promoter regions are known to be an essential mechanism for guiding normal cellular development and play a critical role in the regulation of tumor suppressor gene expression.

Recently, high-resolution methods for genome-wide methylation analysis have been used in the study of HCC, such as in Illumina’s array-based Infinium Methylation Assay [7–10]. These results provide evidence that HCC tumors with specific DNA methylation patterns associated with risk factors or progression of HCC have important clinical applications. In our recent study, we also used a genome-wide methylation array, the Infinium HumanMethylation27 BeadChip to analyze 1968 genes that were hypermethylated in non-tumor tissue and/or tumor tissue from patients with HCC of different viral etiologies.

Molecular signatures causing HCC from chronic infection of HBV or HCV are not clearly known. Some studies suggest that different pathways are preferentially inactivated epigenetically in HCCs caused by different viral infections [18]. To better understand the association between the etiologic-driven changes in DNA methylation patterns and diseases, we use DAVID ontology disease analysis for genes in each etiology. Immune deficiency diseases, including type 1 diabetes, atopy, systemic sclerosis, and cardiovascular diseases were predominantly enriched in HCV-positive HCC, whereas alcoholic diseases and cirrhosis were mainly enriched in HBV-positive HCC. Genes in HCC without hepatitis B and hepatitis C show the association with age-associated diseases and metabolic diseases (Fig. 1b).

The *paired-box* (*PAX*) genes are a family of transcription factors composed of nine members with crucial roles in tissue development, cellular differentiation, migration, and proliferation [19]. *PAX6*, a gene coding for a DNA binding/transcription factor, participates in normal eye, nose, pancreas, and brain development [13]. *PAX6* functions as either oncogene or tumor suppressors which seem to be tissue specific and is discussed controversially [20, 21]. However, methylation promoters of *PAX6* in HCCs have never been illustrated.

In this study, we observed that the *PAX6* expression was frequently down-regulated in HCC cell lines, and the reduced expression is associated with promoter methylation, as confirmed by promoter methylation analyses and pharmacological demethylation treatment. This implicates that DNA methylation is the principle regulatory mechanism of PAX6 inactivation in HCC, suggesting the *PAX6* would be a candidate tumor suppressor in the pathogenesis of HCC. Our findings suggest that hypermethylation of *PAX6* is a common event in HCC but its potential clinical application value needs to be further clarified.

This is the first study to analyze the relationship between *PAX6* methylation status and different viral etiologies in HCC. In accordance with our data, *PAX6* methylation was significantly increased in HBV-positive, HCV-positive, and double-negative HCC tissues as compared with their adjacent non-tumor tissues irrespective of the hepatitis virus status. However, there was no relationship with other clinical characteristics, including age, gender, tumor size, TNM stage, and invasion in HCV-positive HCC (Table 1). Moreover, it was positively associated with invasion in HBV-positive HCC and positively associated with cirrhosis in double-negative HCC tissues. Interestingly, we found that the highest number of methylated genes in HCV-positive HCC tissue (Fig. 1b) and *PAX6* was frequently methylated in HCV-positive HCC tissues (Tables 2 and 3, Fig. 3c). This is consistent with previous studies showing that methylation levels were higher in the HCV-positive HCCs than in the HBV-positive or double-negative HCC [22, 23].

**Table 1:** Clinicopathological characteristics of 160 HCC patients

Characteristics		Cases
Age, year	Mean ± SD	59 ± 14
Gender	Female	94
	Male	66
Hepatitis	HBV-positive	68
	HCV-positive	62
	Double-negative	30
Cirrhosis	No	77
	Yes	80
	Unknown	3
Tumor size, cm	≤3	52
	>3	108
Nodule	Solitary	98
	Multiple	62
AFP level, ng/ml	≤10	45
	>10	113
	Unknown	2
Stage	I	60
	II	46
	III	47
	IV	7
Invasion	No	85
	Yes	75
Recurrence	No	58
	Yes	36
	Unknown	66
Survival	No	71
	Yes	27
	Unknown	62

**Table 2:** Methylation frequency of *PAX6* gene in 349 liver tissues

Etiology	% of methylated cases (methylated cases/total)			*p* value
	Normal controls	Non-tumor tissues	HCC tissues	
All	3.4 % (1/29)	15.6 % (25/160)	39.4 % (63/160)	<0.0001
HBV	10.0 % (1/10)	17.6 % (12/68)	22.1 % (15/68)	0.3243
HCV	0.0 % (0/3)	6.5 % (4/62)	61.3 % (38/62)	<0.0001
NBC	0.0 % (0/16)	30.0 % (9/30)	33.3 % (10/30)	0.0227

Samples with a methylation level above the best cutoff value (MI > 1.13) were determined as methylated cases

**Table 3:** Association between *PAX6* methylation and clinicopathological characteristics of 160 HCC patients

Etiology	All		HBV		HCV		NBC	
	OR	95 % CI	OR	95 % CI	OR	95 % CI	OR	95 % CI
Age	1.04	1.01–1.07	1.03	0.98–1.08	1.02	0.96–1.07	1.02	0.96–1.07
Gender	1.14	0.60–2.16	1.68	0.51–5.48	1.07	0.38–3.00	1.56	0.31–7.87
HBV	0.29	0.15–0.58						
HCV	4.46	2.26–8.80						
Cirrhosis	1.42	0.75–2.69	0.92	0.30–2.78	0.85	0.29–2.50	12.00	1.11–129.42
Tumor size (cm)	0.97	0.90–1.04	0.99	0.89–1.09	0.96	0.81–1.14	1.06	0.89–1.26
Nodules	1.01	0.84–1.20	0.97	0.74–1.27	1.02	0.33–3.11	1.49	0.91–2.44
TNM	1.02	0.72–1.43	1.36	0.74–2.50	0.89	0.48–1.65	1.68	0.75–3.78
AFP	1.24	0.61–2.51	1.17	0.32–4.25	1.46	0.46–4.65	1.00	0.21–4.71
Invasion	1.01	0.56–1.89	2.92	0.90–9.51	0.41	0.14–1.18	2.85	0.57–14.33

*OR* odds ratio, *95 % CI* 95 % confidence interval for the hazard ratio

The HCV core contributes to HCV-mediated carcinogenesis through alteration of various signaling pathways, transcriptional activation, immune modulation, apoptosis, and lipid metabolism [24, 25]. HCV was demonstrated to affect CpG island methylation patterns especially for those genes responsible for DNA mismatch repair (MMR) [26] and cell cycle regulation [27]. Furthermore, core down-regulates the levels of p16 in HCC cells, and via core up-regulates the levels of DNMT1 and DNMT3b to induce promoter hypermethylation [28]. *PAX5*, a member of the PAX family, is frequently inactivated by promoter methylation in HCC and slightly decreases its proliferation rates in HCC cell lines through interaction with a p53 signaling pathway [29]. We therefore reasonably presumed that the HCV core-induced PAX6 methylation consequently contributed to down-regulate PAX6 expression.

In summary, by using genome-wide methylation analysis, we identified the frequently methylated gene *PAX6* in HCC. The quantitative methylation level of *PAX6* was higher in HCC tissues than that in corresponding non-tumor liver tissues irrespective of the virus infection background. In addition, *PAX6* was frequently methylated in HCV-positive HCC tissues. We suggest that the HCV core plays the role of inducing PAX6 methylation. Further investigation is needed to better understand the PAX6 functional pathway in hepatocarcinogenesis.

## Conclusions

Our data suggests that promoter hypermethylation of *PAX6* is a common event in HCCs and the association of *PAX6* methylation in clinicopathological features is divergent with different viral status.

## Methods

### Clinical samples

Twenty-nine specimens of normal livers from hemangiomas, obtained from the Taiwan Liver Cancer Network (TLCN), were used as normal controls in this study. For validation, a total of 160 paired samples of HCC and adjacent non-tumor tissues were used as subjects, including 40 paired samples which were obtained from Tri-Service General Hospital, and 120 paired samples of HCC and adjacent non-tumor tissues were obtained from TLCN. The TLCN is funded by the National Science Council to provide researchers in Taiwan with primary liver cancer tissue specimens and their associated clinical information. The use of clinical samples in this study was approved by our Institutional Review Board and the TLCN User Committee. These specimens were obtained during surgery and were frozen immediately in liquid nitrogen and/or at −80 °C until DNA extraction. The diagnosis of HCC was confirmed by histology. The clinicopathological characteristics of the patients are summarized in Table 1.

### DNA extraction and bisulfite conversion

Genomic DNA from tissues was extracted by using a QIAamp DNA Mini Kit (Qiagen, Hilden, Germany). Genomic DNA extracted from tissues was treated with sodium bisulfite by using an EZ DNA methylation kit (Zymo Research, Orange, CA, USA). For quantitative methylation analysis, CpG methylated human genomic DNA (Thermo Fisher Scientific Inc.) and DNA extracted from normal peripheral blood lymphocytes (PBL) which were modified by sodium bisulfite to generate a positive control and a negative control, respectively.

### Q-MSP

Q-MSP was performed in the TaqMan probe system using the LightCycler 480 system (Roche Applied Science, Mannheim, Germany) and prepared as previously described [12]. Each sample was analyzed in duplicate. The testing results of each sample were assessed as quantification cycle values (Cp value). The *COL2A* gene was used as an internal reference gene by amplifying non-CpG sequences. The results with a Cp value of *COL2A* > 38 were defined as detection failure. The results with a Cp value of *PAX6* > 45 were defined as undetermined after PCR analysis. The DNA methylation level was assessed as a methylation index (MI), using the formula 100 × 2^−[(Cp of *PAX6*) − (Cp of *COL2A*)]^ [30]. The primer and probe sequences are as follows: *COL2A* forward: gggaagatgggatagaagggaatat, reverse: tctaacaattataaactccaaccaccaa, probe: ttcattctaacccaatacct; *PAX6* forward: agggagtatttaatcggttggc, reverse: ctcctacgcctaaaccaaaacg, probe: aaataaaaccgaaccacgatt.

### Statistical analysis

The Wilcoxon rank-sum test and Wilcoxon signed-rank test were used to determine the differences between gene methylation level and disease status. The *χ*^2^ test for trend and logistic regression was used to evaluate the association between gene methylation, disease status, and clinical characteristics. ROC curves were generated to determine the optimal cutoff point of gene methylation for discriminating HCC tissues and normal controls.

## Additional files

Additional file 1: Table S1. Top 50 abnormally methylated genes in HCC with different etiologic factors. (PDF 27.4 kb) Additional file 2: Table S2. Genes in DAVID ontology that abnormally methylated in HBV, HCV, and NBC-HCC. (PDF 6.77 kb)

## Abbreviations

5DAC: 5-aza-2′-deoxycytidine
CGI: CpG islands
HBV: hepatitis B virus
HCC: hepatocellular carcinoma
HCV: hepatitis C virus
PAX6: paired-box 6
Q-MSP: quantitative methylation-specific PCR
TSG: tumor suppressor gene
