# Supplementary material for: Quantitative methylation analysis reveals distinct association between PAX6 methylation and clinical characteristics with different viral infections in hepatocellular carcinoma
Source: Clin Epigenetics. 2016 Apr 22;8:41. doi: 10.1186/s13148-016-0208-3 (PMC4841049; doi:10.1186/s13148-016-0208-3)
Supplement: Additional file 1: Table S1. — Top 50 abnormally methylated genes in HCC with different etiologic factors. (PDF 27.4 kb) [file 13148_2016_208_MOESM1_ESM.pdf]

Table S1. TOP50 abnormally methylated genes in HCC with different etiologic factors.

Etiologic factors	Methylation in HCC	TOP	SYMBOL
All	HYPER	1	DLX5
All	HYPER	2	ALX4
All	HYPER	3	WT1
All	HYPER	4	EYA4
All	HYPER	5	MSX1
All	HYPER	6	KCNQ1
All	HYPER	7	ALDH1A3
All	HYPER	8	BNC1
All	HYPER	9	CDKN1C
All	HYPER	10	NEUROG1
All	HYPER	11	PTGS2
All	HYPER	12	RUNX3
All	HYPER	13	SYK
All	HYPER	14	APC
All	HYPER	15	CALCA
All	HYPER	16	HOXA9
All	HYPER	17	HOXB4
All	HYPER	18	PYCARD
All	HYPER	19	SOCS2
All	HYPER	20	TWIST1
All	HYPER	22	BCL2
All	HYPER	23	DCC
All	HYPER	24	GSTP1
All	HYPER	25	HOXD4
All	HYPER	26	ISYNA1
All	HYPER	27	KCNQ1DN
All	HYPER	28	PTPRO
All	HYPER	29	RASSF5
All	HYPER	30	SEMA3B
All	HYPER	31	SMPD3
All	HYPER	32	TNFRSF10C
All	HYPER	38	BTG4
All	HYPER	39	HSPA2
All	HYPER	40	MYOD1
All	HYPER	41	OVOL1
All	HYPER	42	RBP1
All	HYPER	43	ACTG1
All	HYPER	44	ADAM33
All	HYPER	45	ADAMTSL2
All	HYPER	46	ADCY2
All	HYPER	47	ADCY4
All	HYPER	48	ADCY5
All	HYPER	49	ADRB1
All	HYPER	50	AJAP1
All	HYPO	1	ATP10A
All	HYPO	2	GNAS
All	HYPO	3	IGF2AS

All	HYPO	4	INS
All	HYPO	5	ADAM2
All	HYPO	6	ANKRD7
All	HYPO	7	C1orf42
All	HYPO	8	C21orf129
All	HYPO	9	C5orf20
All	HYPO	10	CABP5
All	HYPO	11	CCL1
All	HYPO	12	CCL20
All	HYPO	13	CD72
All	HYPO	14	CRNN
All	HYPO	15	CST4
All	HYPO	16	DCD
All	HYPO	17	DEFB103A
All	HYPO	18	DEFB123
All	HYPO	19	DEFB4
All	HYPO	20	DNAJB8
All	HYPO	21	ELA2
All	HYPO	22	FCN2
All	HYPO	23	FGF6
All	HYPO	24	FLG
All	HYPO	25	FLJ00060
All	HYPO	26	FLJ14816
All	HYPO	27	FLJ43806
All	HYPO	28	GML
All	HYPO	29	GRM4
All	HYPO	30	GRM8
All	HYPO	31	GZMB
All	HYPO	32	H1T2
All	HYPO	33	HAS1
All	HYPO	34	IVL
All	HYPO	35	K6HF
All	HYPO	36	K6IRS3
All	HYPO	37	KRT1
All	HYPO	38	KRT16
All	HYPO	39	KRT2A
All	HYPO	40	KRT2B
All	HYPO	41	KRT6C
All	HYPO	42	KRTHA4
All	HYPO	43	KRTHB5
All	HYPO	44	LYZL4
All	HYPO	45	MARCO
All	HYPO	46	MFAP5
All	HYPO	47	MSR1
All	HYPO	48	NALP10
All	HYPO	49	NALP14
All	HYPO	50	NPBWR2
B+C	HYPER	1	GALR1
B+C	HYPER	2	CCND2

B+C	HYPER	3	CHFR
B+C	HYPER	4	CRABP1
B+C	HYPER	5	CCNA1
B+C	HYPER	6	DLK1
B+C	HYPER	7	PRG2
B+C	HYPER	8	SNRPN
B+C	HYPER	9	TFPI2
B+C	HYPER	10	ALS2CR11
B+C	HYPER	11	C3orf52
B+C	HYPER	12	CD200
B+C	HYPER	13	CDK5R2
B+C	HYPER	14	CIDEA
B+C	HYPER	15	CPLX2
B+C	HYPER	16	CRISPLD1
B+C	HYPER	17	CX36
B+C	HYPER	18	DAB1
B+C	HYPER	19	DDAH2
B+C	HYPER	20	DEGS2
B+C	HYPER	21	DMRT1
B+C	HYPER	22	ENPP2
B+C	HYPER	23	FLJ45983
B+C	HYPER	24	FMNL1
B+C	HYPER	25	FXYD3
B+C	HYPER	26	GFPT2
B+C	HYPER	27	GFRA3
B+C	HYPER	28	GHSR
B+C	HYPER	29	GPR103
B+C	HYPER	30	GPR6
B+C	HYPER	31	GRIA4
B+C	HYPER	32	HAND2
B+C	HYPER	33	HDAC3
B+C	HYPER	34	HOXA4
B+C	HYPER	35	HOXC11
B+C	HYPER	36	HOXC5
B+C	HYPER	37	HOXD3
B+C	HYPER	38	IGSF9
B+C	HYPER	39	IL4I1
B+C	HYPER	40	KCNA3
B+C	HYPER	41	KCNK3
B+C	HYPER	42	KCNN3
B+C	HYPER	43	LAMA2
B+C	HYPER	44	LOC349136
B+C	HYPER	45	LRAT
B+C	HYPER	46	LTF
B+C	HYPER	47	MAP4K1
B+C	HYPER	48	MGC39545
B+C	HYPER	49	MPP2
B+C	HYPER	50	MSX2
B+C	HYPO	1	C11orf39

B+C	HYPO	2	C15orf32
B+C	HYPO	3	C1QB
B+C	HYPO	4	CDSN
B+C	HYPO	5	DBH
B+C	HYPO	6	F2
B+C	HYPO	7	FOXI1
B+C	HYPO	8	GNMT
B+C	HYPO	9	KRTHA2
B+C	HYPO	10	LZTS1
B+C	HYPO	11	NR0B2
B+C	HYPO	12	OLFM4
B+C	HYPO	13	OR6A2
B+C	HYPO	14	PDZK1
B+C	HYPO	15	PRDM11
B+C	HYPO	16	SEC31L2
B+C	HYPO	17	TGM5
B+C	HYPO	18	TRIM31
B+C	HYPO	19	ZNF423
B+C	HYPO	20	KRTAP8-1
B+C	HYPO	21	RETNLB
B+C	HYPO	22	SMCP
B+C	HYPO	23	OR2C3
B+C	HYPO	24	REG3A
B+C	HYPO	25	GRIP1
B+C	HYPO	26	IFNA8
B+C	HYPO	27	KRTAP15-1
B+C	HYPO	28	CMKLR1
B+C	HYPO	29	REG1B
B+C	HYPO	30	TOLLIP
B+C	HYPO	31	MARCH1
B+C	HYPO	32	KRTHA1
B+C	HYPO	33	EPN3
B+C	HYPO	34	TNFRSF10A
B+C	HYPO	35	MYH6
B+C	HYPO	36	KRTHA6
B+C	HYPO	37	KRTAP13-3
B+C	HYPO	38	HCRTR2
B+C	HYPO	39	PRSS2
B+C	HYPO	40	SERPINA12
B+C	HYPO	41	SIGLEC12
B+C	HYPO	42	TGIF2LY
B+C	HYPO	43	PDPK1
B+C	HYPO	44	PLCL1
B+C	HYPO	45	TBX6
B+C	HYPO	46	LOC348645
B+C	HYPO	47	GYPE
B+C	HYPO	48	DYRK2
B+C	HYPO	49	DEFB126
B+C	HYPO	50	CUBN

B+N	HYPER	1	SFRP1
B+N	HYPER	2	ACTN2
B+N	HYPER	3	ADAM12
B+N	HYPER	4	BRSK2
B+N	HYPER	5	C11orf45
B+N	HYPER	6	C1orf51
B+N	HYPER	7	C3orf15
B+N	HYPER	8	CBR3
B+N	HYPER	9	CD40
B+N	HYPER	10	CHST4
B+N	HYPER	11	CTSF
B+N	HYPER	12	D4S234E
B+N	HYPER	13	DIRAS2
B+N	HYPER	14	DKFZP586H2123
B+N	HYPER	15	EPHA7
B+N	HYPER	16	FLJ42486
B+N	HYPER	17	GULP1
B+N	HYPER	18	HIST1H1A
B+N	HYPER	19	HRH3
B+N	HYPER	20	KCNN2
B+N	HYPER	21	KIAA1944
B+N	HYPER	22	LY75
B+N	HYPER	23	ME1
B+N	HYPER	24	MYO3A
B+N	HYPER	25	NELL2
B+N	HYPER	26	NOTCH3
B+N	HYPER	27	NPBWR1
B+N	HYPER	28	OCIAD2
B+N	HYPER	29	OLIG3
B+N	HYPER	30	PLD5
B+N	HYPER	31	PRKG1
B+N	HYPER	32	PROK2
B+N	HYPER	33	PTF1A
B+N	HYPER	34	SFRP4
B+N	HYPER	35	SLC13A5
B+N	HYPER	36	SLC18A3
B+N	HYPER	37	SLC2A11
B+N	HYPER	38	SMOC2
B+N	HYPER	39	SOX11
B+N	HYPER	40	SPSB4
B+N	HYPER	41	ST6GALNAC3
B+N	HYPER	42	TRPM3
B+N	HYPER	43	ULBP3
B+N	HYPER	44	WDR17
B+N	HYPER	45	BCAT2
B+N	HYPER	46	EED
B+N	HYPER	47	FRMD5
B+N	HYPER	48	FLJ38725
B+N	HYPER	49	STK25

B+N	HYPER	50	THBS4
B+N	HYPO	1	ADORA3
B+N	HYPO	2	BPIL3
B+N	HYPO	3	C11orf38
B+N	HYPO	4	CCKAR
B+N	HYPO	5	DIRAS3
B+N	HYPO	6	FLJ14346
B+N	HYPO	7	GPR109A
B+N	HYPO	8	HK2
B+N	HYPO	9	IL17E
B+N	HYPO	10	K5B
B+N	HYPO	11	KRT6E
B+N	HYPO	12	LILRB5
B+N	HYPO	13	MGC27016
B+N	HYPO	14	OR12D3
B+N	HYPO	15	PAQR4
B+N	HYPO	16	SURF5
B+N	HYPO	17	UBD
B+N	HYPO	18	C16orf44
B+N	HYPO	19	FLJ33860
B+N	HYPO	20	CNKSR1
B+N	HYPO	21	FLJ25801
B+N	HYPO	22	NDUFAF1
B+N	HYPO	23	TNFRSF4
B+N	HYPO	24	COMT
B+N	HYPO	25	IFNB1
B+N	HYPO	26	SIGLEC6
B+N	HYPO	27	LILRA4
B+N	HYPO	28	FLJ14668
B+N	HYPO	29	TRPM8
B+N	HYPO	30	ZNF532
B+N	HYPO	31	UBQLN3
B+N	HYPO	32	S100A12
B+N	HYPO	33	PTPRS
B+N	HYPO	34	QTRT1
B+N	HYPO	35	MX2
B+N	HYPO	36	POLR3D
B+N	HYPO	37	ZNF572
B+N	HYPO	38	TPSB2
B+N	HYPO	39	PSG3
B+N	HYPO	40	PCP4
B+N	HYPO	41	LY6D
B+N	HYPO	42	ATXN3
B+N	HYPO	43	APOBEC3A
B+N	HYPO	44	SEC61A2
B+N	HYPO	45	CLEC10A
B+N	HYPO	46	TRY1
B+N	HYPO	47	PSMD6
B+N	HYPO	48	ARHGEF18

B+N	HYPO	49	PDZRN4
B+N	HYPO	50	CHRM1
C+N	HYPER	1	ZMYND10
C+N	HYPER	2	ESR1
C+N	HYPER	3	LOX
C+N	HYPER	4	ANKRD33
C+N	HYPER	5	ARNT2
C+N	HYPER	6	BIK
C+N	HYPER	7	BMP6
C+N	HYPER	8	CACHD1
C+N	HYPER	9	CPXM2
C+N	HYPER	10	DKFZp434N062
C+N	HYPER	11	DNAH3
C+N	HYPER	12	ELN
C+N	HYPER	13	EMX2
C+N	HYPER	14	EVX1
C+N	HYPER	15	FGF19
C+N	HYPER	16	FGF20
C+N	HYPER	17	FGFR2
C+N	HYPER	18	FLJ12505
C+N	HYPER	19	FLJ14001
C+N	HYPER	20	FOXE1
C+N	HYPER	21	GNG4
C+N	HYPER	22	GPR135
C+N	HYPER	23	HKR1
C+N	HYPER	24	HSF4
C+N	HYPER	25	IRF6
C+N	HYPER	26	KIAA0980
C+N	HYPER	27	KIRREL2
C+N	HYPER	28	LOC342897
C+N	HYPER	29	MAPK13
C+N	HYPER	30	MGC42105
C+N	HYPER	31	MMP2
C+N	HYPER	32	MYADM
C+N	HYPER	33	NPM2
C+N	HYPER	34	NRIP2
C+N	HYPER	35	PITX2
C+N	HYPER	36	PLEK2
C+N	HYPER	37	PPT2
C+N	HYPER	38	PRAC
C+N	HYPER	39	RAC2
C+N	HYPER	40	RBP7
C+N	HYPER	41	SCARF2
C+N	HYPER	42	SHOX2
C+N	HYPER	43	SUSD1
C+N	HYPER	44	SYNPO2
C+N	HYPER	45	TINAGL1
C+N	HYPER	46	TLR2
C+N	HYPER	47	TLX2

C+N	HYPER	48	TRIM59
C+N	HYPER	49	TRPV4
C+N	HYPER	50	UAP1L1
C+N	HYPO	1	MEST
C+N	HYPO	2	ATP6V0D2
C+N	HYPO	3	CALN1
C+N	HYPO	4	DNAHL1
C+N	HYPO	5	EDG7
C+N	HYPO	6	FAM19A3
C+N	HYPO	7	HLA-DRA
C+N	HYPO	8	ISG20L2
C+N	HYPO	9	KRTHB4
C+N	HYPO	10	LACRT
C+N	HYPO	11	MGC11257
C+N	HYPO	12	MGC13034
C+N	HYPO	13	PAEP
C+N	HYPO	14	PLXNA4B
C+N	HYPO	15	PRB4
C+N	HYPO	16	SBEM
C+N	HYPO	17	SCN7A
C+N	HYPO	18	SGCD
C+N	HYPO	19	SLIC1
C+N	HYPO	20	TMEM86B
C+N	HYPO	21	TPO
C+N	HYPO	22	TRPV6
C+N	HYPO	23	WDR21C
C+N	HYPO	24	ZP4
C+N	HYPO	25	C15orf2
C+N	HYPO	26	ZNF445
C+N	HYPO	27	C18orf16
C+N	HYPO	28	MYT1
C+N	HYPO	29	LGI1
C+N	HYPO	30	RALGDS
C+N	HYPO	31	OCM
C+N	HYPO	32	HRH2
C+N	HYPO	33	PRB2
C+N	HYPO	34	SLC2A5
C+N	HYPO	35	CORO7
C+N	HYPO	36	SLC10A6
C+N	HYPO	37	MRPL55
C+N	HYPO	38	CASP8
C+N	HYPO	39	MGC7036
C+N	HYPO	40	GDF5
C+N	HYPO	41	LOC161931
C+N	HYPO	42	TRIM42
C+N	HYPO	43	SCN4A
C+N	HYPO	44	SSNA1
C+N	HYPO	45	TNFSF11
C+N	HYPO	46	CASP1

C+N	HYPO	47	KRTHB3
C+N	HYPO	48	DLG2
C+N	HYPO	49	C1orf36
C+N	HYPO	50	MRGPRX2
HBV	HYPER	1	ADAM23
HBV	HYPER	2	C18orf34
HBV	HYPER	3	C1QTNF1
HBV	HYPER	4	C6orf155
HBV	HYPER	5	CAMK4
HBV	HYPER	6	CART
HBV	HYPER	7	CD44
HBV	HYPER	8	CTNND2
HBV	HYPER	9	DPYSL4
HBV	HYPER	10	ERBB4
HBV	HYPER	11	FAM84A
HBV	HYPER	12	HS3ST2
HBV	HYPER	13	HTR1B
HBV	HYPER	14	KCNH4
HBV	HYPER	15	KCNH8
HBV	HYPER	16	KCNQ3
HBV	HYPER	17	KCNV1
HBV	HYPER	18	LRFN5
HBV	HYPER	19	MAL
HBV	HYPER	20	MGC46496
HBV	HYPER	21	PLAC2
HBV	HYPER	22	RLN2
HBV	HYPER	23	SKIP
HBV	HYPER	24	SUSD3
HBV	HYPER	25	SYT10
HBV	HYPER	26	TRIM38
HBV	HYPER	27	TSPAN2
HBV	HYPER	28	UNQ739
HBV	HYPER	29	VGLL2
HBV	HYPER	30	WDR52
HBV	HYPER	31	SLIT2
HBV	HYPER	32	HLXB9
HBV	HYPER	33	PEX5L
HBV	HYPER	34	FLRT2
HBV	HYPER	35	KIT
HBV	HYPER	36	RYR2
HBV	HYPER	37	BACE2
HBV	HYPER	38	SLC9A3
HBV	HYPER	39	AMPH
HBV	HYPER	40	IGFBP7
HBV	HYPER	41	SLIT3
HBV	HYPER	42	GRIK1
HBV	HYPER	43	MICA
HBV	HYPER	44	GRIK2
HBV	HYPER	45	ALK

HBV	HYPER	46	DUSP26
HBV	HYPER	47	P4HA3
HBV	HYPER	48	ST8SIA2
HBV	HYPER	49	ADAMTS18
HBV	HYPER	50	OPRK1
HBV	HYPO	1	CLEC4M
HBV	HYPO	2	IGHG3
HBV	HYPO	3	IQCF2
HBV	HYPO	4	MSLN
HBV	HYPO	5	PRDM2
HBV	HYPO	6	SERPINF1
HBV	HYPO	7	SLC24A5
HBV	HYPO	8	TMC2
HBV	HYPO	9	PNLIPRP2
HBV	HYPO	10	ASB16
HBV	HYPO	11	PPP2R2C
HBV	HYPO	12	ANGPT4
HBV	HYPO	13	MMP3
HBV	HYPO	14	IFNA4
HBV	HYPO	15	TUBB4
HBV	HYPO	16	LCE1E
HBV	HYPO	17	MOG
HBV	HYPO	18	DARC
HBV	HYPO	19	CCL13
HBV	HYPO	20	CRISPLD2
HBV	HYPO	21	PSG4
HBV	HYPO	22	NEUROD6
HBV	HYPO	23	CRHR1
HBV	HYPO	24	NTNG2
HBV	HYPO	25	C10orf39
HBV	HYPO	26	PSG5
HBV	HYPO	27	BCAR1
HBV	HYPO	28	ITGBL1
HBV	HYPO	29	CNOT6
HBV	HYPO	30	LOC120379
HBV	HYPO	31	SRP9
HBV	HYPO	32	KCTD17
HBV	HYPO	33	PARVG
HBV	HYPO	34	NRM
HBV	HYPO	35	Pfs2
HBV	HYPO	36	MUC5AC
HBV	HYPO	37	MT4
HBV	HYPO	38	LCE2B
HBV	HYPO	39	HLA-DQB2
HBV	HYPO	40	ENDOGL1
HBV	HYPO	41	DEFB125
HBV	HYPO	42	CLEC12B
HBV	HYPO	43	CASQ2
HBV	HYPO	44	LPIN2

HBV	HYPO	45	PVALB
HBV	HYPO	46	KRT5
HBV	HYPO	47	PSG9
HBV	HYPO	48	L2HGDH
HBV	HYPO	49	FLJ38379
HBV	HYPO	50	SCGB1D1
HCV	HYPER	1	C19orf30
HCV	HYPER	2	ACCN4
HCV	HYPER	3	AVPR1B
HCV	HYPER	4	AXL
HCV	HYPER	5	CECR6
HCV	HYPER	6	CLIC6
HCV	HYPER	7	COL1A2
HCV	HYPER	8	COL23A1
HCV	HYPER	9	CRH
HCV	HYPER	10	CSRP1
HCV	HYPER	11	FBLIM1
HCV	HYPER	12	FZD6
HCV	HYPER	13	HOXB5
HCV	HYPER	14	HOXD10
HCV	HYPER	15	HR
HCV	HYPER	16	IPF1
HCV	HYPER	17	JAZF1
HCV	HYPER	18	KCNA2
HCV	HYPER	19	KCNS3
HCV	HYPER	20	LYL1
HCV	HYPER	21	NPC1L1
HCV	HYPER	22	OCA2
HCV	HYPER	23	PDE8B
HCV	HYPER	24	PDIA2
HCV	HYPER	25	ST6GALNAC2
HCV	HYPER	26	TRAPPC1
HCV	HYPER	27	UBTD1
HCV	HYPER	28	UNQ3045
HCV	HYPER	29	CRYGN
HCV	HYPER	30	GSTM1
HCV	HYPER	31	MTL5
HCV	HYPER	32	RALGPS1
HCV	HYPER	33	SLC6A4
HCV	HYPER	34	PAQR5
HCV	HYPER	35	C6orf134
HCV	HYPER	36	CHGA
HCV	HYPER	37	DCHS1
HCV	HYPER	38	C10orf35
HCV	HYPER	39	CHAC1
HCV	HYPER	40	TCF7
HCV	HYPER	41	IL12RB2
HCV	HYPER	42	COL4A3
HCV	HYPER	43	RPL26L1

HCV	HYPER	44	NES
HCV	HYPER	45	DGCR6L
HCV	HYPER	46	GDPD5
HCV	HYPER	47	A4GALT
HCV	HYPER	48	PAX6
HCV	HYPER	49	HSP90AB1
HCV	HYPER	50	FAM57B
HCV	HYPO	1	AGT
HCV	HYPO	2	AKAP3
HCV	HYPO	3	ANKRD30A
HCV	HYPO	4	C12orf46
HCV	HYPO	5	DPPA3
HCV	HYPO	6	ESRRB
HCV	HYPO	7	FLJ45202
HCV	HYPO	8	HBII-438B
HCV	HYPO	9	IL1F5
HCV	HYPO	10	IL1F9
HCV	HYPO	11	LY9
HCV	HYPO	12	PCDHGC5
HCV	HYPO	13	SLC35C1
HCV	HYPO	14	CD79A
HCV	HYPO	15	MGC16291
HCV	HYPO	16	OR2A4
HCV	HYPO	17	TOP1MT
HCV	HYPO	18	SPINK1
HCV	HYPO	19	C1orf38
HCV	HYPO	20	TNNT3
HCV	HYPO	21	CNR2
HCV	HYPO	22	SLC18A1
HCV	HYPO	23	NYD-SP26
HCV	HYPO	24	DEFB105A
HCV	HYPO	25	HEMGN
HCV	HYPO	26	ZNF610
HCV	HYPO	27	EPB41L1
HCV	HYPO	28	CALCB
HCV	HYPO	29	GJB4
HCV	HYPO	30	ADCY9
HCV	HYPO	31	BTN2A1
HCV	HYPO	32	MLLT11
HCV	HYPO	33	NALP5
HCV	HYPO	34	RASGRP3
HCV	HYPO	35	ADAM18
HCV	HYPO	36	MMP7
HCV	HYPO	37	FGL1
HCV	HYPO	38	DEFA3
HCV	HYPO	39	S100A10
HCV	HYPO	40	ZNF137
HCV	HYPO	41	S100A5
HCV	HYPO	42	SYNGR2

HCV	HYPO	43	PIK3R5
HCV	HYPO	44	LCP2
HCV	HYPO	45	KRTAP19-3
HCV	HYPO	46	SLC5A12
HCV	HYPO	47	MAP3K7IP1
HCV	HYPO	48	JMJD4
HCV	HYPO	49	ANK2
HCV	HYPO	50	PCDH12
NBC	HYPER	1	CTSZ
NBC	HYPER	2	SFRP5
NBC	HYPER	3	C7orf13
NBC	HYPER	4	CKB
NBC	HYPER	5	DDR2
NBC	HYPER	6	DNAJC6
NBC	HYPER	7	DYRK1B
NBC	HYPER	8	FLJ32130
NBC	HYPER	9	GPR75
NBC	HYPER	10	MT1E
NBC	HYPER	11	PYY
NBC	HYPER	12	SEPT10
NBC	HYPER	13	TIMP4
NBC	HYPER	14	TMEM42
NBC	HYPER	15	UPK3B
NBC	HYPER	16	ZP3
NBC	HYPER	17	ALS2CL
NBC	HYPER	18	MGC35308
NBC	HYPER	19	DUSP2
NBC	HYPER	20	ECHDC3
NBC	HYPER	21	CENTG2
NBC	HYPER	22	IFT57
NBC	HYPER	23	SNF1LK
NBC	HYPER	24	IVNS1ABP
NBC	HYPER	25	HIST1H1D
NBC	HYPER	26	VLDLR
NBC	HYPER	27	TCF15
NBC	HYPER	28	PRDM16
NBC	HYPER	29	GATA5
NBC	HYPER	30	CAMTA2
NBC	HYPER	31	CDCA7
NBC	HYPER	32	GPR24
NBC	HYPER	33	HSU79303
NBC	HYPER	34	ARL10
NBC	HYPER	35	VAX2
NBC	HYPER	36	LENG9
NBC	HYPER	37	CDX2
NBC	HYPER	38	DUSP23
NBC	HYPER	39	HIST1H3H
NBC	HYPER	40	GSTM3
NBC	HYPER	41	MICAL-L2

NBC	HYPER	42	ZBTB16
NBC	HYPER	43	GPR137B
NBC	HYPER	44	ANGPTL4
NBC	HYPER	45	LMOD1
NBC	HYPER	46	C3orf57
NBC	HYPER	47	VDR
NBC	HYPER	48	CENTA2
NBC	HYPER	49	SSBP4
NBC	HYPER	50	SLC9A2
NBC	HYPO	1	GPR45
NBC	HYPO	2	PHYHIP
NBC	HYPO	3	SLC17A4
NBC	HYPO	4	SYNC1
NBC	HYPO	5	TAS2R60
NBC	HYPO	6	TMEM129
NBC	HYPO	7	CYP11B2
NBC	HYPO	8	TAGAP
NBC	HYPO	9	CDH5
NBC	HYPO	10	TXNL6
NBC	HYPO	11	MYL1
NBC	HYPO	12	OR2F1
NBC	HYPO	13	SNAPAP
NBC	HYPO	14	MKRN3
NBC	HYPO	15	UGT1A1
NBC	HYPO	16	SLC39A12
NBC	HYPO	17	SLC3A1
NBC	HYPO	18	TSPAN32
NBC	HYPO	19	FAM83F
NBC	HYPO	20	PI15
NBC	HYPO	21	FHL2
NBC	HYPO	22	KRT6B
NBC	HYPO	23	PRAME
NBC	HYPO	24	MS4A1
NBC	HYPO	25	MC3R
NBC	HYPO	26	TNFSF12-TNFSF13
NBC	HYPO	27	ATP4A
NBC	HYPO	28	ROS1
NBC	HYPO	29	CNGA1
NBC	HYPO	30	C20orf79
NBC	HYPO	31	NDUFA8
NBC	HYPO	32	FAM49A
NBC	HYPO	33	FLJ33706
NBC	HYPO	34	FOLR2
NBC	HYPO	35	S100A1
NBC	HYPO	36	FLJ46481
NBC	HYPO	37	FLJ31222
NBC	HYPO	38	HTR3C
NBC	HYPO	39	HK3
NBC	HYPO	40	CSRP3

NBC	HYPO	41	STYXL1
NBC	HYPO	42	UGT8
NBC	HYPO	43	GPR109B
NBC	HYPO	44	PLA2G2D
NBC	HYPO	45	S100A13
NBC	HYPO	46	PPP3R2
NBC	HYPO	47	C16orf30
NBC	HYPO	48	RBP3
NBC	HYPO	49	CNGA3
NBC	HYPO	50	HBD
