# Supplementary material for: Quantitative methylation analysis reveals distinct association between PAX6 methylation and clinical characteristics with different viral infections in hepatocellular carcinoma
Source: Clin Epigenetics. 2016 Apr 22;8:41. doi: 10.1186/s13148-016-0208-3 (PMC4841049; doi:10.1186/s13148-016-0208-3)
Supplement: Additional file 2: Table S2. — Genes in DAVID ontology that abnormally methylated in HBV, HCV, and NBC-HCC. (PDF 6.77 kb) [file 13148_2016_208_MOESM2_ESM.pdf]

Table S2. Genes in DAVID ontology that abnormally methylated in HBV, HCV, and NBC-HCC.

Etiology	Term	Genes
HBV	alcohol abuse	CRHR1, GABRG2, HTR1B, GRIN2B, APOE, GABRB2, OPRK1
	cirrhosis, alcoholic; alcoholism	GABRG2, GRIN2B, GABRB2, CRHR1,
	schizophrenia	GABRG2, ERBB4, GRIK1, GRIK2, GABRB2, GRIK3, TH, ST8SIA2, ALK, MOG, DMPK, GAD2, HTR1B, GRIN2B, DARC, APOE, CHRNA7, TCF4
HCV	diabetes, type 1	TCF7, CALD1, CTLA4, ACP1, CD48, IL12RB2, SLC11A1, CYBA, ACE, GCK, AGT, PON1, NOS3, NQO1, IGFBP2, LTA, LAG3, IGFBP5
	cardiovascular	CYBA, F12, ACE, PTGIS, AGT, SLC6A4, PON1, NOS3
	atopy	IL12RB2, SLC11A1, ACE, AGT, CTLA4, MS4A2, LTA, MIF
	systemic sclerosis	ACE, COL1A2, CTLA4, NOS3, CYP2E1
	nephropathy, IgA	CYBA, ACE, SERPINB7, PON1, NOS3, ST6GALNAC2, MBP
	aplastic anemia, acquired	GSTM1, CTLA4, CYP2E1, NQO1
NBC	macular degeneration, age-related	CFH, ELAVL4, VLDLR, CKB
	blood pressure, arterial hypertension	CAV1, CYP11B2, CFH, SLC4A1
